# Understanding patient-related barriers to hydroxyurea use among adolescent and adult patients with sickle cell disease in Mulago and Kiruddu hospitals, Uganda, a qualitative study

**DOI:** 10.1186/s12913-024-11125-6

**Published:** 2024-05-27

**Authors:** Priscilla Namaganda, Patience Nantume, Kelvin Roland Mubiru, Adelliine Twimukye, Christine Sekaggya Wiltshire

**Affiliations:** 1 Kiruddu National Referral Hospital, P.O. Box 6553, Kampala, Uganda; 2 Infectious Disease Institute, Makerere University, Kampala, Uganda; 3 Uganda Cancer Institute, Kampala, Uganda

**Keywords:** Sickle cell disease, Hydroxyurea, Patient-related barriers.

## Abstract

**Background:**

In 2016, Uganda added Hydroxyurea (HU) to the list of essential drugs to treat sickle cell disease SCD. However, Hydroxyurea utilization has been low for several countries in sub-Saharan Africa. This study examined patient-related barriers to hydroxyurea use among adolescent and adult patients with sickle cell disease in Mulago and Kiruddu hospitals, in Uganda.

**Methods:**

To understand the patient-related barriers to hydroxyurea use among adolescent and adult patients with sickle cell disease, we conducted a parallel convergent mixed methods study at outpatient departments of two national referral hospitals in Uganda from October 2022 to January 2023. The cross-sectional mixed-methods study employed both quantitative and qualitative methods. We collected survey data from a systematic sample of 259 participants and conducted individual interviews with a purposive sample of 40 participants (20 adolescents or their caregivers and 20 adult patients with SCD) and interviewed them individually on their knowledge, perceptions, barriers, and facilitators of HU utilization. Descriptive data were analyzed using Stata 16, whereas qualitative data were analyzed thematically using an inductive approach supported by NVivo 12 software. We triangulated data to determine the concordance of qualitative and quantitative data.

**Results:**

The study enrolled 40 participants for qualitative interviews and 259 patients for quantitative, with an average age of 16, over half being female, 46% having secondary education, and 96% unmarried. The prevalence of HU use was 78%. The study identified three themes as follows: Patient barriers at the individual including Inadequate knowledge about HU, Persistent pain, Poor adherence to HU, Poor communication with health care workers, and Psychosocial and emotional challenges. At the facility level, long queues and poor quality of care, drug-related side effects that affect HU, and drug stock-outs were reported. Myths, rumors, and misconceptions about HU, and gender-related barriers were reported to affect HU utilization at a community level. Facilitators for the use of HU and recommendations for improvement. Facilitators included perceived benefits, long duration on HU, information sharing by healthcare workers, availability of complementary drugs, confirmation of diagnosis, and availability of medication at public health facilities or private pharmacies. Patients suggested continuous adherence support, encouragement from healthcare workers, sensitization about benefits and risks, a peer-to-peer approach, and financial support for adolescents and women to start businesses to resolve financial problems.

**Conclusion:**

Implementing the use of HU has been challenging in Uganda and needs improvement. Facilitators to hydroxyurea use have been highlighted, though Patient-identified barriers at individual, facility, and community levels that need to be resolved. The experiences and insights shared by our participants provide invaluable guidance for increasing the uptake of HU. Further studies are needed to establish validated instruments to assess patients’ pain communication and adherence to the HU regimen.

## Background

In Africa, sickle cell disease (SCD) contributes substantially to mortality in children younger than 5 years. The global burden has been quantified, with SCD accounting for 6.4% of the under-5 mortality across all of Africa [1]. However, in countries with greater sickle allele frequencies and lower childhood mortality rates, such as Uganda, SCD may account for up to 15% of under-5 mortality [2]. The mortality rate in adult patients with SCD is not known presumably because of a lack of accurate data but is thought to be high as more children with SCD survive into adolescence and adulthood, they are faced with poor access to comprehensive sickle cell care with a continuing risk of complications or death [2]. 

Hydroxyurea is one of the approved drugs for treating sickle cell disease [3]. The mechanism by which hydroxyurea works is rather unknown although its efficacy in the treatment of SCD is generally attributed to its ability to boost the levels of fetal hemoglobin (Hb F, α_2_γ_2_) hence lowering the concentration of HbS. HbF is protective against clinical severity, and low-percentage HbF is associated with a higher risk of developing Vaso-occlusive complications, organ damage, and early death. Systemic review studies have documented the efficacy of hydroxyurea in adult patients with SCD [3]. In Uganda, the NOHARM and REACH studies reported a reduction in SCD-related complications with the use of hydroxyurea and appeared to be safe for children with SCD without increased severe malaria, infections, or adverse events [4, 5].

Hydroxyurea was added to the list of essential drugs in Uganda in 2016 but it is not readily available [6].

The number of patients with SCD currently taking hydroxyurea is about 33%; this is undocumented data from patient charts. This low percentage of use could be due to limited access and availability of drugs, practitioners’ low knowledge of HU use, and patients’ fear of drug toxicities. Therefore, this study sought to assess barriers to HU treatment among this cohort of patients and document them. This study focused on knowledge, perceptions, barriers, and facilitators of adolescent and adult patients with sickle cell disease regarding HU because there is minimal data and even the data that is available focuses on children, not adults. Reasons reported by authors in studies done included fear of cancer and other side effects, not wanting to take a medication, not wanting to have required laboratory monitoring, or not thinking the medication would work [7]. The primary goal and benefit of patient-centered care is to improve individual health outcomes, not just population health outcomes, although population outcomes may also improve [8]. Not only do patients benefit, but providers and healthcare systems benefit as well, through (a) Improved satisfaction among patients and their families, (b) Enhanced reputation of providers among healthcare consumers, (c) Better morale and productivity among clinicians and ancillary staff, (d) Improved resource allocation, (e) Reduced expenses and increased financial margins throughout the continuum of care [8]. 

With greater use of HU for eligible patients, it is expected that fewer patients will be hospitalized for complications of SCD, resulting in a net reduction of national healthcare costs for patients with SCD [9]. In addition, the broader appropriate use of HU in patients with SCD should improve their quality of life and productivity [9]. With this information, we can lobby the government and/or donors to avail recourses for continued supply of HU and other resources like laboratory capacity that aid us in giving comprehensive care to patients with SCD. Therefore, we conducted a mixed methods study to identify the knowledge, perceptions, barriers, and facilitators of adolescent and adult patients with sickle cell disease regarding HU and suggest interventions to facilitate HU uptake in Uganda. We hypothesized that there was a relationship between patient-related barriers and hydroxyurea use among patients with SCD.

## Methods

### Study design

From October 2022 to January 2023, we conducted a cross-sectional mixed-methods study (parallel convergent) with qualitative components using a phenomenological approach.

### Study setting

The study was conducted at the Sickle cell clinic in Mulago Hospital sickle cell clinic and Kiruddu Hospital, hematology. The Sickle Cell Clinic is an innovation for treating children with SCA with acute pain and other medical complications as outpatients. The Mulago sickle cell clinic attends to children, adolescents, and adult patients with SCD. The Kiruddu clinic attends to adolescent and adult patients with SCD in addition to other hematological conditions. Both Mulago and Kiruddu are national referral hospitals in Uganda and are teaching hospitals for Makerere University, College of Health Sciences. The Mulago SCD clinic cares for more than 300 patients with SCD. The hematology clinic at Kiruddu Hospital cares for 100 to 150 patients with SCD.

### Sample size estimation

We purposively selected 40 participants to participate in the qualitative interviews. The actual sample size (40) was determined by how many participants were required to explore all the research questions and to achieve thematic saturation. It was difficult to determine the ideal sample size for achieving these objectives at the early stage of the research. Therefore, the process of participant selection was iterative, involving several rounds of selection and interviews as will be necessary to achieve thematic saturation. Data was collected until no new themes or patterns emerged from participants interviewed participants selected from each study site. 20 individual interviews were carried out in each selected site, making a total of 40 Individual interviews from two study sites. Patients were interviewed to identify the barriers to the use of hydroxyurea through In-depth interviews using an in-depth interview form developed for the study with 20 purposively selected patients per site. The criteria for the selection of patients for in-depth interviews were 10 adults (5 males and 5 females), and 10 adolescents (5 males and 5 females) who have ever missed appointments or drugs from each clinic. Also, their understanding, experiences, and what they had heard regarding using Hydroxyurea were assessed. All the interviews were conducted from the hospital premises and in a language preferred by the participants and audio recorded. All adolescents were interviewed with their caretakers and each caretaker signed a parent-guardian consent form in addition to the adolescent signing an assent form.

Eligibility for HU use was (a) Three or more sickle cell-associated moderate to severe pain crises in 12 months, requiring hospitalization or management at a health facility, (b) Sickle cell-associated pain that interferes with daily activities and quality of life, (c) History of severe and/or recurrent acute chest syndrome, (d) Severe symptomatic chronic anemia that interferes with daily activities or quality of life(severe symptomatic anemia criteria was assessed based on the need for blood transfusion). if participants responded yes to any of the above criteria, they were eligible for HU use. Patients with other sickle cell syndromes – e.g., Hb SC disease, S/ß thalassemia, pregnant, severely ill study, and declined to participate in the study were excluded.

The sample size for quantitative was estimated using Leslie Kish’s (1964) formula for sample size calculation. With a prevalence of HU use at 33.7% as reported by a study done in Oman [10] and at a 0.05 level of significance, the sample was estimated at 260 participants. The sickle cell clinic at Mulago Hospital runs daily and that at Kiruddu on Thursday of every week. Patients with SCD who came for assessment were screened using a questionnaire developed for the study and each one of them was informed about the study with the help of a research assistant. Patients who are taking HU or have taken HU were recorded. Patients who are not taking HU were assessed to determine if they fit the criteria for starting HU as described above. If participants responded to any of the above criteria, they were enrolled in the study after obtaining informed consent from research assistants. Patients enrolled were asked to fill out a standardized questionnaire with the help of research assistants. Information obtained included (a) demographic i.e. age, gender, address, level of education, religion, and occupation, (b) time when patient joined the clinic, past and current medications, history of SCD-associated complications and history of admissions, (c) status of HU use and reasons for not initiating HU and possible solutions to these challenges.

### Study variables

#### Independent variables

We collected data on; age, gender, address, level of education, religion, occupation, commonest complications of SCD experienced, indications for HU use, and the common medications used.

#### Dependent variables

Our outcomes were willingness to use HU, perceptions about HU use, reasons for not initiating HU and possible solutions to these challenges.

### Procedures for data collection and instruments

#### Quantitative data

We used a systematic sampling method for the survey. For the quantitative objective, all patients with SCD were screened and those who met the inclusion criteria were enrolled in the study. We therefore included every 4th participant in the survey beginning with the 4th adult until the sample size was attained. We used maximum variation purposive sampling to select the participants for the in-depth interviews. For the survey, we used an interviewer-administered semi-structured questionnaire to collect data on the 259 participants using a questionnaire administered by a research assistant. For the 40 individual interviews, we used an interview guide which was used to collect perceptions on HU. Interviews lasted approximately 10–20 min. Data collection occurred over three months and transcription began as data collection was ongoing.

#### Data quality control

The questionnaire was pre-tested on 5 participants from the same community to ensure that the questions were clear and understandable to participants. The Questionnaires and Interview guide were translated into the local language and then back-translated to English as part of standard operating procedures such that they have retained their meaning. The research assistants were adequately trained for 7 days and routinely supervised while in the field and the data they were collecting by the principal investigator to ensure the correct use of data collection tools and adherence to ethical principles.

To ensure reliability, we set clear research questions to expand on responses. Qualitative data was collected separately from quantitative data (parallel convergence). Codes and qualitative findings were crosschecked to improve reliability. Consensus between two or more observers was done to establish reliability. We used NVivo version 12 software to manage narrative data.

To ensure validity, all transcripts were checked for accuracy and completeness by the interviewers to enhance data validity. Feedback from research participants (member check) after analysis and interpretation was obtained in an organized results dissemination workshop. Documentation of member checks and interpretations that were changed because of member feedback was done. Triangulation combined quantitative (survey) and qualitative data collection methods (in-depth interviews) in this single study. Triangulation of various data collection methods was used. These included questionnaires, Topic guides (In-depth interviews), transcripts, field notes, and Literature review. This was aimed at verifying information, or facts obtained from using other methods.

#### Data management and analysis

Quantitative data collected were double-entered into the computer using EPI-DATA (version 3.1) software to minimize data entry errors. Data was exported to STATA version 15 for data cleaning and analysis. Data was then backed up and archived using codes to ensure confidentiality. The descriptive characteristics were presented using frequencies and percentages or proportions in tables. Numerical data was summarized using means and standard deviations for normally distributed continuous data or medians and interquartile ranges for continuous but skewed variables. The prevalence of hydroxyurea use among adult patients was calculated as a proportion of adult patients with SCD who have ever used hydroxyurea out of the total number of participants who are eligible for HU use with its 95% confidence intervals.

The Qualitative study was guided by the ethical principles of the Association of Social Anthropologists. These principles included protecting research participants, anticipating harm, avoiding undue intrusion, rights to confidentiality and anonymity, intellectual property rights, and participant involvement in research. Recording and storing participants’ information was done in a manner that facilitated greater confidentiality and anonymity, including the use of pseudonyms to describe participants during interviews, separation of participants’ ID information from their transcripts, storage of participant information in secured locations and password-protected hard drives, removal of participants names in all research dissemination outputs. Research assistants obtained informed consent prior to start of individual interviews. The Individual interviews were conducted in one-to-one and face-to-face format to provide greater privacy and assure participants of confidentiality. We conducted an inductive thematic analysis collected from individual interviews with different respondent categories such as (20 adolescents or their caregivers and 20 adult patients with SCD). The analysis examined meanings, themes, and patterns that manifested texts from the interviews regarding HU use in two hospitals in Uganda. All audio recordings from open-ended questions based on interview guides were transcribed verbatim. Two coders Individually read each transcript line by line and identified key concepts to develop a coding framework. A coding framework based on eight transcripts that were manually reviewed and coded to generate the initial set of codes that were crosschecked iteratively between two coders (AT & PN) for consensus and to improve reliability. All transcripts were imported into NVivo version 12 software for open coding and management of data. An initial codebook was developed, and the revised codes were grouped into categories and identified themes. Illustrative quotations for each emergent theme were selected for the results narration.

## Results

**Fig. 1:**
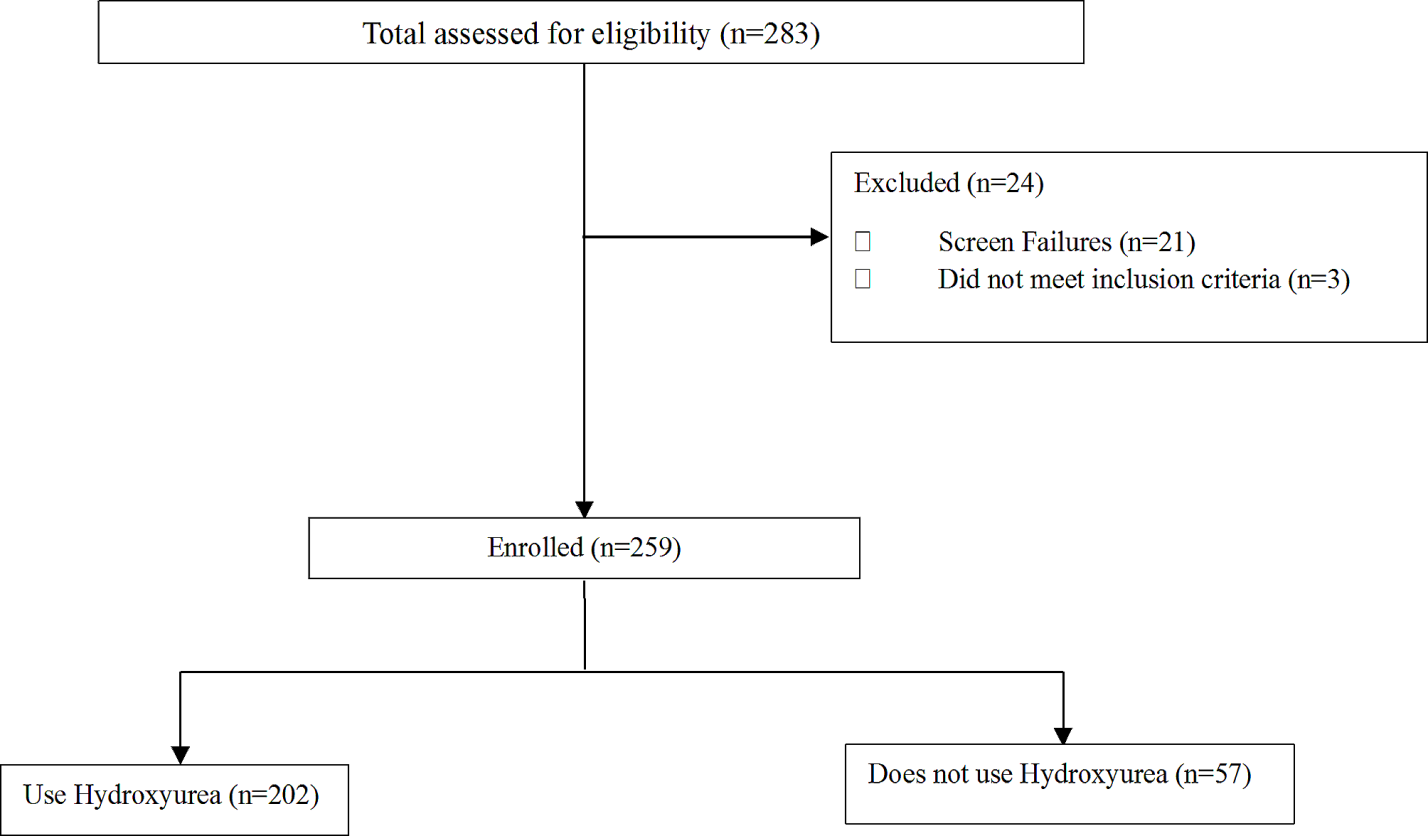
Flow chart showing enrolment profile

283 participants were assessed and 259 were enrolled in the study as shown in the flow chart, Fig. 1.

### Characteristics of adults and adolescents

The total sample size was 259, 221 participants were recruited from the Mulago Hospital sickle cell clinic and 38 from Kiruddu Hospital. At the Mulago sickle cell clinic, the average age was 16 years, with 58.5% being female, and 46% having an education level of secondary. At Kiruddu Hospital, the median age was 24, with 71.1% being female, and 48.1% having an education level of secondary. The average cost of transport to and from the hospital was 11,000 Ugx. The average duration of HU use was 24 months. All this is summarized in Table 1.

**Table 1:** Social demographic characteristics of study participants in the hydroxyurea (HU) in Kiruddu and Mulago hospitals in Uganda

	Mulago Hospital (*N* = 221)	Kiruddu Hospital (*N* = 38)	Total (*N* = 259)
**Age**			
Median (Q1,Q3)	16.0 (14.0, 20.0)	24.0 (19.2, 28.0)	17.0 (14.0, 20.0)
**Gender**			
Male	91 (41.2%)	11 (28.9%)	102 (39.4%)
Female	130 (58.8%)	27 (71.1%)	157 (60.6%)
**Education Level**			
Primary level	95 (43.0%)	12 (31.6%)	107 (41.3%)
Secondary level	101 (45.7%)	23 (60.5%)	124 (47.9%)
Tertiary level	23 (10.4%)	2 (5.3%)	25 (9.7%)
Not educated	2 (0.9%)	1 (2.6%)	3 (1.2%)
**Marital Status**			
Cohabiting	2 (0.9%)	0 (0.0%)	2 (0.8%)
Married	0 (0.0%)	2 (5.3%)	2 (0.8%)
Not Married/Single	212 (95.9%)	34 (89.5%)	246 (95.0%)
Separated	6 (2.7%)	2 (5.3%)	8 (3.1%)
Widowed	1 (0.5%)	0 (0.0%)	1 (0.4%)
**Type of person interviewed**			
Adults	87 (39.4%)	35 (92.1%)	122 (47.1%)
Caregivers + adolscents	134 (60.6%)	3 (7.9%)	137 (52.9%)
**Transport Cost to and From the Clinic (Ugx Shillings)**			
Median (Q1,Q3)	12000.0 (8000.0, 16000.0)	10000.0 (6000.0, 15750.0)	11000.0 (8000.0, 16000.0)

### Eligibility for HU use

259 participants met the criteria for HU, 202(78.0%) were taking HU, and 57 participants met the criteria for HU use but were not taking HU. Of those that use HU, 92.1% were current users and 7.9% were past users. HU use among eligible patients is shown in Table 2.

**Table 2:** HU use among eligible patients

	Overall (*N* = 259)
Have you used hydroxyurea?	
Yes	202 (78.0%)
No	57 (22.0%)
**If yes**,	
Past	16 (7.9%)
Present	186 (92.1%)

### Patient-related barriers to HU use

Painful crisis was the most common indication for HU use reported in 94.6% of participants, followed by chest syndrome (28%), anemia, 19.4%, and avascular necrosis, 24.7%. indication for HU use is summarized in Table 3 above.

**Table 3:** Summary of Indications for initiating HU use

	Mulago Hospital (*N* = 158)	Kiruddu Hospital (*N* = 28)	Total (*N* = 186)
How long have you been taking hydroxyurea for (Years)			
Median (Q1,Q3)	2.0 (1.0, 3.0)	1.1 (0.4, 2.0)	2.0 (0.9, 3.0)
**Current dose (in milligrams)**			
Median (Q1,Q3)	1000.0 (1000.0, 1000.0)	1000.0 (1000.0, 1000.0)	1000.0 (1000.0, 1000.0)
**Indications of HU use**			
Pain Crisis	153 (96.8%)	23 (82.1%)	176 (94.6%)
Chest Syndrome	36 (22.8%)	16 (57.1%)	52 (28.0%)
hypertension	4 (2.5%)	2 (7.1%)	6 (3.2%)
Anaemia	26 (16.5%)	10 (35.7%)	36 (19.4%)
SCD Kidney disease	2 (1.3%)	1 (3.6%)	3 (1.6%)
Avascular necrosis	30 (19.0%)	16 (57.1%)	46 (24.7%)
Leg Ulcers	0 (0.0%)	1 (3.6%)	1 (0.5%)
Priapism	0 (0.0%)	2 (7.1%)	2 (1.1%)
Cardiac/Heart complications	1 (0.6%)	2 (7.1%)	3 (1.6%)
Gallstones/cholelithiasis	0 (0.0%)	0 (0.0%)	0 (0.0%)
Stroke	4 (2.5%)	2 (7.1%)	6 (3.2%)

### Indications for initiating HU use

The study identified three themes as follows: Patient barriers at the individual, facility, and community level as shown in Tables 4, 5 and 6, and 7. Facilitators for the use of HU and recommendations for improvement as displayed in Table 8.

**Table 4:** Theme and sub-themes of barriers to hydroxyurea use among adolescent and adult patients with sickle cell disease in Uganda. Source: (Researchers own work)

Theme	Sub-themes
Barriers	
1. Individual patient	1.1. Financial constraints 1.2. Inadequate knowledge about HU 1.3. Persistent pain 1.4. Poor adherence to HU 1.5. Poor communication with health care workers 1.6. Psychosocial and emotional challenges
2. Facility related	1.1. Long queues and poor quality of care 1.2. Perceived drug-related side effects that affect HU. 1.3. drug stock-outs;
3. Community, Social cultural barriers	3.1. Myths, rumors, and misconception about HU 3.2. Gender-related barriers affecting the utilization of HU

**Table 5:** Reasons for stopping HU use

	Mulago Hospital (*N* = 13)	Kiruddu Hospital (*N* = 3)	Total (*N* = 16)
**How long did you take Hydroxyurea for (Months)**			
Median (Q1,Q3)	2.0 (1.0, 6.0)	12.0 (6.2, 18.0)	3.0 (0.9, 8.0)
Can’t afford to buy the medicine	10 (76.9%)	3 (100.0%)	13 (81.2%)
Experienced side effects with the medicine	2 (15.4%)	0 (0.0%)	2 (12.5%)
Felt better so I didn’t think I needed it any more	0 (0.0%)	0 (0.0%)	0 (0.0%)
Did not feel better or did not experience any benefits from the medicine	0 (0.0%)	0 (0.0%)	0 (0.0%)
Too many pills to take	1 (7.7%)	0 (0.0%)	1 (6.2%)

**Table 6:** Knowledge of HU and its benefits

	Overall (*N* = 262)
**Treatment for SCD**	
Yes	231 (88.2%)
No	31 (11.8%)
**Treatment of cancer**	
Yes	0 (0.0%)
No	262 (100.0%)

**Table 7:** Theme and sub-themes of facilitators to hydroxyurea use among adolescent and adult patients with sickle cell disease in Uganda. Source: (Researchers own work)

Theme	Sub themes
Facilitators	
1. Individual patient	1.1. Hydroxyurea use was described as an effective pain relief and symptom control. 1.2. HU improves quality of life. 1.3. Enhances ability to do daily activities or tasks. 1.4. Regulated the blood levels and reduced the level of the disease. 1.5. HU prevented anemia, stroke, and other complications. 1.6. Enhanced patient confidence
2. Facility related	2.1. Awareness and information shared about HU. 2.2. Health education and seminars held about HU. 2.3. Complementary drugs used along HU. 2.4. Confirmation of diagnosis for sickle cell prior to HU initiation 2.5. Availability of HU drugs

**Table 8:** ; Themes and subthemes on recommendations from participants on how to alleviate barriers to SCD care. Source: (Researchers own work)

Theme	Sub themes
Recommendations	
1. Individual patient	1.1. Patients should receive continuous advice and encouragement from healthcare workers. 1.2. Patients need to be told about the benefits of HU use. 1.3. Patients need to have continuous sensitization about the benefits and risks of HU use. 1.4. Patients need to be counselled on HU use as well as the value of life. 1.5. A peer-to-peer approach using experienced patients to encourage and support each other needs to be adopted. 1.6. There is need for financial support to start a business as a source of income to cater for medical needs.
2. Facility related	2.1. Healthcare workers should address the fears and rumors about sick cell disease and treatment. 2.2. Healthcare workers should advise/encourage patients to adhere to sickle cell medication. 2.3. There should be free drugs availed at the health facilities. 2.4. Health care workers should do follow-ups of patients and set up reminder mechanisms to adhere to treatment and keep routine clinic visits. 2.5. Health facilities should allow credit options for patients who lack funds to buy drugs.
3. Family	3.1. Financial empowerment for women to support sickle cell children at the family level should be done
4. Community	4.1. Extension of sickle cell health services to the communities and local districts should be done to resolve the issue of transport. 4.2. Sensitization of patients and community people about Hydroxyurea should be done.
5. National	5.1. Government should reduce the price of sickle cell medicine to enhance access for all patients. 5.2. The government should put up sickle cell health facilities in rural areas. 5.3. There is a need for a collaborative partnership with international or non-governmental organizations to support sickle cell patients.

### Individual barriers

#### Financial constraints

The most common reason for stopping HU use was lack of affordability reported by 81.2% of participants. This information is summarised in Table 5 below. The major challenge among 19 (48%) participants from qualitative interviews was financial constraints to buy medication, and food and meet the transport costs to access care.

*“There are times when I skipped my medicine doses… Sometimes it was due to money; if I didn’t have the money to buy the tablets. Because we are supposed to buy the tablets—sometimes you go to the pharmacy, and they are expensive, yet you don’t have money. You use the little money you must buy some tablets and they get finished, and I have no money to buy more. So, I first miss some days, and a few times I depend on Panadol and Ibuprofen for emergencies. I take it in case I don’t have the medicine not daily—they told me I could take it occasionally if I am constrained financially and unable to buy the medicine”.*

-- IDI, 18, female, adult, Mulago.

Most adults and adolescents were not able to pay for prescribed sickle cell drugs and other ailments because they were expensive.

*“… There was no money to buy it because a packet is shs.15,000; just one packet… yet buying it is expensive and I have no money. Now I no longer stay with my father; my mother stays home; she does not work. If my father goes to work and takes a long time there, my mother is suffering to buy the medicine. She buys it for shs. 15,000 but it gets finished in a week, so she must look for more shs. 15,000 to buy another dose”.*

-- IDI, 19, Male, adolescent, Mulago.

Most of the adolescents said they were unemployed and were mainly dependent on their parent’s support, which was sometimes limited:

*“If there is transport because I am always at home. Ever since they fired me from my job, I have been at home”.*

-- IDI, 3 1, female, adolescent, Mulago.

### Inadequate knowledge

Inadequate knowledge about HU among adolescents and adults by few participants. Generally, most individual participants from qualitative (27, 68%) and quantitative (231 (88.2%) across all age categories had a high level of awareness about hydroxyurea and its related benefits as shown in Table 5 below. For the few adult participants who lacked awareness about Hydroxyurea, it was because health workers did not inform them about it, and some had never started on it.

*Because I didn’t know about it [Hydroxyurea]; they had never told me about it. So, the doctors asked me, “Have you ever used hydroxyurea?” and I told them, “I don’t know about it*.

-- IDI, 22, male, adult, Mulago.

Among adolescents, there was low knowledge about HU due to limited sensitization as they did not receive enough health education. Moreover, they reported rumors, myths, and misconceptions about sickle cell in the community.

*“Most times what scares them, they say, “Once you start taking the drug and then stop you die”, it scares them as well—okay, the rumors are so many”.*

-- IDI, 11, Male, youth, Mulago.

Some participants said there was a lack of awareness about Hydroxyurea in the community because they were not sensitized and had not come across people who swallowed it:

*“Community people have not been well oriented about Hydroxyurea benefits by the doctors… they have not heard anyone on it so far”.*

-- IDI, 16_ adult, Mulago.

### Persistent pain affected the use of HU

Nearly half (38%) of the participants who participated in individual interviews reported persistent pain despite taking painkillers as a major challenge that hindered proper adherence.

*They used to administer painkillers to me since I had a lot of pain! And if the pain comes, it is so strong; it is severe because at school there is a health facility they would do their best with the painkillers but they had no effect. So, they would bring me here”*.

-- IDI, 28, female, youth, Mulago.

Some participants said they could hardly walk to the health facility due to severe pain, hence missed appointments and picking drugs.

I missed my appointment because I was sick and bedridden, admitted to the hospital. Huh! I feel pain. There are times I could hardly walk….

-- IDI, 10, female, adult, Mulago.

### Poor adherence to HU

43% (17, 43%), mainly adolescents from qualitative interviews reported Poor treatment adherence. They missed taking sickle cell medication on time or stopped it for a month or more. The major reasons for missing drugs were pain, running out of drugs, missed appointments to pick drug refills, and lack of funds to buy drugs when they ran out:

*“So, when I swallow it in the morning, it takes like 30 minutes, then I go back to normal. But sometimes I miss doses because the medicine is finished before the people at home bring for me more, because I am not near home. They bring it within one week because they also must look for the money to buy it.*

-- IDI, 28, female, adolescent, Mulago.

Another reason for poor adherence to sick cell medication was forgetfulness. Some participants said they came back from school tired and slept off while others could be distracted by television:

*“We leave preps at around 9 pm and I must wash my uniform, I must do this or that. So after, you are exhausted and you just fall on the bed; maybe you say, “Let me read this and swallow the medicine, then I sleep” but before you realize it, they are ringing the bell to wake students up. So that is when I have forgotten and then I remember”.*

--IDI, 28, female, adolescent, Mulago.

One participant said they had poor access to medication when traveling due to the loss of a loved one and suffered too much pain. They also missed appointments due to illness and admission.”

*“I had lost someone. Sometimes, I would be ill and have a lot of pain. I felt too much pain. I missed a dose… Still, I have ever missed taking a dose that was because of my illness. I was admitted”.*

--IDI, 9, male, adult, Mulago.

Some participants missed drugs due to academic reasons. They could not leave school during the exam period to pick treatment refills.

*“There is a time I missed some appointments that in turn made me miss drugs…I am in boarding school and my appointment was due during my examination period, so I was unable to come”.*

-- IDI, 19, Male, adolescent, Mulago).

One caretaker said adolescent was tired of swallowing several tablets daily hence missed taking drugs on some days.

*“Talking about being fed up with the medicine; sometimes I say[caregiver], “I will give you shs.500 if you[adolescent] swallow the medicine”. So personally, when I noticed that on the weekend, she swallows 2000 mg, I said, “Okay, let us leave Monday” and we went against the doctor’s instructions. So honestly, we gave Monday a break; we don’t swallow medicine because she swallowed 2 tablets on Sunday and 2 tablets on Saturday, so we rested on Monday. We resume swallowing it on Tuesday till Sunday, then we rest on Monday. But eh! She totally didn’t like it! So, we discussed and came to that agreement, so that is how we do it”.*

-- IDI, 30, female, caregiver, Mulago.

***Missed routine clinic visits or appointments affected adherence.***

Most participants [11] missed routine clinic visits or appointments mainly because they lacked money for transport to the health facility. The cost of transport for some participants was Ugx 20,000.

*“I have never missed a clinic appointment and you said yes…I missed because my mother did not have money for transport, yet she had debts—she had to work so that she could pay the debt she had”.*

--IDI, 21, female, adolescent, Mulago.

Missed appointments hindered participant’s access to medical care and routine sickle cell monitoring.

*“Ever since we started this medication, they told us not to miss any appointments because she must come back and monitor her progress ever since she started hydroxyurea. I believe we won’t miss it again. Previously, we could buy our own medicine but this time round, we won’t miss clinic appointments again”.*

-- IDI, 14, caregiver, female, Mulago.

Transport constraints were the major reasons for missed appointments. Some participants said they lived at far-off distances from health facilities.

*“Since sometimes I live far away and sometimes there is no money for the transport fare… Because there are times when I am suffering symptoms of the illness and I have no transport to bring me to the health Centre for instance now, I just borrowed the money to bring me here. So, I fall sick and swallow the medicine, then I feel better”.*

-- IDI, 31, female, adult, Mulago.

Only one participant reported that a busy work schedule hindered appointment-keeping.

*“I miss appointments because sometimes I am weak, or sometimes I might be busy. I am healthy, but the work I am doing keeps me busy. That is what usually happens”.*

-- IDI, 22, male, adult, Mulago.

### Psychosocial, and emotional challenges

Some participants said they lacked a positive outlook on life. They emphasized they lacked motivation, zeal, and interest to continue taking HU drugs because they had lost hope for a complete cure for sickle cell:

*“Sometimes, we lack the zeal to come since we do not have the hope to get completely cured but if the doctor tells you to keep coming and follows you up and encourages you”* --IDI, 10, female, adult, Mulago.

Some participants (n = 11, 28%) were anxious and worried to take drugs for life without stopping as they worried could cause undesirable side effects:

*“For us that have used it, we experience the benefits, but also worry about the risks like kidney, failure because those are the crucial body organs! So, we worry about all that” --.* IDI, 30, female, adult, Mulago.

Some participants (38%) from individual interviews suffered stress due to severe pain especially when they missed medication. They felt bad when missed sickle cell drugs because it would result in painful episodes.

*“What I know is that it [hydroxyurea] prevents the painful episodes or controls it but now if you don’t swallow it, the pain returns and this time it is severe. That is what I’m scared of”.*

--IDI, 20, male, youth, Mulago.

### Perceived drug-related side effects affected

Some participants [10] experienced drug-related side effects that affected adherence to HU, such as headache, dizziness, Painful erections at night, frequent urination, and eyes turned yellow.

*“I would have headaches every single day without a break. So, there was a health worker we asked while I was admitted, and he said this drug does not cause headaches and we ignored the issue. But it was a severe headache; they first stopped me from taking it for some time… And they [doctors] told her to stop taking it for 2 weeks and that’s what she did”.*

--IDI, 24, female, adolescent, and caregiver, Mulago.

Some participants were anxious and worried about taking drugs for life without stopping as they worried could cause undesirable side effects:

*“For us that have used it, we experience the benefits, but also worry about the risks like kidney, failure because those are the crucial body organs! So, we worry about all that”.*

--. IDI, 30, female, adult, Mulago.

### Facility-related barriers

#### Long queues

Long queues that led to poor quality of care were also reported in the hospital. Some participants were concerned about doctors who left without attending to them, yet they were in severe pain.

“*Anha! Sometimes you go and they tell you, “The time is up; the doctor is leaving, the patients’ queue was long”, sometimes you go and there is no medicine”.*

-- IDI, 5, female, adult, Mulago.

Some participants said there was no counseling support at the health facility regarding sickle cell at the health facility:

“Uh, they [doctors] did not support me when I missed my appointment. When I came, I was registered and given treatment. They did not do anything else or give me any form of counseling”.

*--IDI, 9, male, adult, Mulago*.

#### Drug stock-outs

Drug stock-outs were mainly reported by adults. lack of sickle cell medication and other drugs in public health facilities leads patients to run out of drugs. They were told to buy the prescribed drugs from other private pharmacies to resolve issues of drug stock-outs, yet they could not afford them.

*“About a month. Initially, they would give us three or six sachets of medicine. They can give you medicine for a two months or three months dose. Then, I buy the rest in the pharmacy. The challenge is some pharmacies do not have it in stock”.*

-- IDI, 10, female, adult, Mulago.

Some participants reported a lack of free medication in public hospitals.

*“…Because even if you find someone and say, “Please help me with shs. 30,000 to buy medicine” they tell you, “Go to Mulago, medicine is there free of charge!” But they don’t know the problem is you will get there and fail to get it”.*

-- IDI, 5, female, adult, Mulago.

#### Poor communication with health providers

One participant said they did not report drug-related side effects they experienced to healthcare workers due to forgetfulness.

*“I forgot to tell the health workers about the side effects I suffered. Sometimes I simply forget all about it and ignore it and say, “As long as it [side effect] is over, I continue swallowing…Now these health workers initiated me into that medicine and told me to swallow it. I would never skip a dose. They told me to swallow it from Monday to Friday, then I take a different dose for the weekend, and then it is the same for the following week”.*

*--* IDI, 19, Male, Youth, Mulago.

#### Community-based barriers

Some participants reported that rumors, myths, and misconceptions were the major concerns about sickle cell in the community.

*“Most times what scares them, they say, “Once you start taking the drug and then stop you die”, it scares them as well—okay, the rumors are so many”* -- IDI, 11, Male, youth, Mulago.

Some participants said there was limited access to sickle cell medication in their nearby community health facilities:

*“One time my leg was in severe pain; I had not yet seen the doctor. So, if the health facility was nearby—at that time I would go to Nakaseke, and they work on me. But now, there are no health workers who handle sickle cell cases in Namuwogga”.*

*--* IDI, 27, female, adult Mulago.

Family disagreements or conflict arises from lack of financial provision by man for woman to take child to hospital:

*“Sometimes we get challenges, and it affects the family. We even get disagreements between the mother and father; sometimes you are supposed to take the child and he says, “I have no money! If you can go, then go. If you are unable to go, you will go some other time” yet the appointment date is due. So, those are some of the challenges we experience” --*IDI, 26, female, caregiver Mulago.

### Facilitators for the use of HU

Facilitators reported by participants were mainly at the Individual and facility level as displayed in Table 7.

### Individual level

Positive perception of HU among adolescents and adults facilitated its utilization. Hydroxyurea use was described as effective pain relief, symptom control, and good and helpful treatment among most of the qualitative participants (n = 28, 70%). This was because it mainly relieved and prevented painful sickle cell episodes, stroke, and malaria. One participant said she received pain relief having taken hydroxyurea, she also read via a Google search about weight gain benefits related to hydroxyurea’s use.

*“I suffered severe pain before you started taking it [Hydroxyurea]. The pain was severe! It was so strong that sometimes I could faint because the pain was overwhelming…So far, it is not a bad medicine; it has helped us to decrease the pain so that it is not severe”.*

-- IDI, 3, female, adolescent, Kiruddu.

Patients reported that Hydroxyurea use improves quality of life as it reduces opportunistic infection and frequenting of hospitals by patients.

*“The medicine can make you not go to the hospital every day because the day I began taking the medicine, I stopped going to the hospital. I stopped having infections. The medicine can protect you from getting sick in the cold. You can do anything even when you do not put on a sweater. You feel better. Even if you feel pain, it is not so much”-*.

- IDI, 15, adult male, Mulago.

Some participants illustrated the ability to do daily activities or tasks very well after using hydroxyurea. They said they had the energy to wash, cook, and perform house chores. Hydroxyurea uses enhanced mobility as one participant said could travel on long journeys having taken it.

*“Initially, my back would hurt whenever I would walk a distance such as from this place to home which is no longer the case. I am better. I can perform my tasks very well. I wash, cook, and do everything very well”.*

--IDI, 9, male, adult, Mulago.

Participants said hydroxyurea use regulated the blood levels and reduced the level of the disease in the body or the blood.

*“It seems when we had just gotten initiated onto hydroxyurea, the body was not yet used to it, so the blood levels were a bit unstable. Because when we initiated her onto hydroxyurea, they first checked her blood levels and all that. When we had just initiated her, her blood levels went down, and they initiated her back onto the drug around 2018 in December. And ever since that time, we have not had challenges with her blood levels, expect just eating lots of avocado to supplement”.*

-- IDI, 30, female, caregiver, Mulago.

Patients reported that Hydroxyurea use prevents a child from anemia, stroke, and other complications.

“Those [patients in waiting area] I have heard them say that it has worked for them; those I have heard especially while we are seated. They say it was effective for them; if the child had many pain episodes, they reduced. Or if the blood levels were reduced—even my brother; he used to have anemia and the pain was severe but when he started swallowing it, it started to reduce”.

--IDI, 24, female, caregiver, Mulago.

Long duration on HU, enhanced patient confidence. Most of the participants were aware of hydroxyurea because they had experienced taking it for more than a year which enhanced their confidence. Duration on medication among most participants was between one to eleven years, few took it for less than a year.

*“I started hydroxyurea this year in January; we came for a check-up after I went to school because I’m in boarding. Now usually when you take hydroxyurea—remember it is daily, every day I must— “*.

--IDI, 28, female, adolescent, Mulago.

### Facility level

Information shared by healthcare workers about HU enhanced its uptake. Health providers prescribed hydroxyurea and shared information about it through health education and seminars. They taught about the benefits and risks, the importance of hydroxyurea, and the need for patients to alert doctors in case they experienced unusual Side effects.

*“…for us, we got to learn about the side effects from the doctors. But while we were at the seminar, they taught us that it is under the supervision of the doctors, who check frequently. But when we gather as women, those who know and those who don’t know, they say, “It burns the liver and the kidneys”, others say, “It is expensive”. But since you have some knowledge about it, you are firm and whatever you notice about it, you inform the doctor, “Doctor, I notice my fingers are turning” and they counsel you. Because even if we are taking it, as parents we are worried”.*

-- IDI, 30, female, adult, Mulago.

Healthcare workers performed testing to confirm the diagnosis of sickle cell before treatment initiation that enhanced treatment prescription. There was only one participant who said was started on treatment after symptom identification such as joint and leg swelling.

*“I was then diagnosed with sickle cells. So, they asked them, “What signs have you noticed?” Then they said that they saw my joints and legs swelling. So, they got to understand and prescribe medication”.*

-- IDI, 10, female, adult, Mulago.

The availability of HU drugs enhanced its uptake. Some participants said they bought sickle cell medication from private pharmacies or clinics whenever drugs were not available at public health facilities.

*“They [doctors from public facility] gave me the prescription and told me to buy it. So, I bought and took it from the private pharmacy, it was finished. So, after a while, I bought more medicine, but they told us we had to swallow it every day”.*

-- IDI, 5, female, adult, Mulago.

**Recommendations from participants on how to alleviate barriers to SCD care**

### Individual

The major recommendation to patients was for patients to receive continuous advice and encouragement from healthcare workers. They particularly desired health workers to remind them about perfectly adhering to Hydroxyurea because it reduced the constant attacks and kept them healthy. They could advise them to set up reminders such as alarms for perfect adherence.

*“We need to receive advice from the health workers. Sometimes, we lack the zeal to come since we do not have the hope to get completely cured but if the doctor tells you to keep coming and follows you up and encourages you”* --IDI, 10, female, adult, Mulago.

Patients need to be told about the benefits of hydroxyurea, so that they may accept its early initiation and follow the doctor’s instructions.

*“In order to accept the drug; we must tell them about the advantages of hydroxyurea. And I think we should also give them examples; I don’t know if you the health workers see that—personally, my child; I know that when she started taking hydroxyurea, her health condition became better. I regretted why they didn’t tell me earlier, but I feared it because I heard them say, “Once she gets initiated onto it, she takes it for life” and I would say, “Argh!” I was quite afraid of it. But I realized that in this life, many people are taking medicine daily and it helps them. [Hmmm] Maybe Musawo, the question I want to ask you today is, if the person grows up and gets to the child-bearing age, does she stop taking hydroxyurea”.*

*--* IDI, 26, female, caretaker, Mulago.

Continuous sensitization about the benefits and risks of hydroxyurea, to create awareness.

*“Other people need more teaching about the thing, and telling them, “If the child takes it, it will reduce the number of times you come here due to the child’s severe health condition”*-.

- IDI, 28, female, Youth, Mulago.

Counseling hydroxyurea should be done as well as counseling patients to value life.

*“A way of encouraging people is to educate them about how important their health is; he shouldn’t be discouraged because he is neither the first nor last because there are many people out there who are ill, but everyone must believe in themselves so that they are healthy. So, if my parent cares, I also must care for myself to make sure I am healthy for the sake of my parent, instead of saying, “I don’t want to swallow medicine” one must sacrifice!*

--IDI, 18, female, adult, Mulago.

A peer-to-peer approach using experienced patients encourages patients to support each other and testify about the benefits of adhering to sickle cell treatment.

*“…there is a school with children who suffer from sickle cell who are about 10. So, they asked me for advice, “Should we swallow hydroxyurea?” because I have spent 7 years. And I told them, “You swallow it, I also swallow it” I don’t discourage them because I have grown up without hydroxyurea. But I said the young ones know that it is helpful to them, so I cannot tell them about the fingernails turning black. Because I see their fingernails; they are not as black as I teach them different vocational skills in their school and they ask me, “Do you also swallow this medicine?” and I tell them I swallow it. But I do notice them.*

-- IDI, 27, female, adult Mulago.

Need financial support to start up a business as a source of income for medication to meet transport, food, and other needs.

*“I was thinking, since she has finished Senior 6, maybe I could start up a small business for her so that she earns some money. Even if she gets shs. 2000, she saves it for clinic appointments and buying medicine”* --IDI, 24, female, youth, Mulago.

Urge patients to drink plenty of water and mind their diet and clothing.

*“They [patients] should be cautious of the cold weather by wearing a scarf. You should take water instead of Soda, minute maid [ soft drink] because that is not recommended. You would rather buy passion fruits and make your juice since the other drinks contain acids that are not good. I would rather drink water instead of drinking those other drinks. [Okay! ] You must wear a sweater when it is cold and desist from drinking cold water in the cold weather. You must mix it with hot water to become warm”.*

-- IDI, 17, female, Mulago.

### Health facility based

Healthcare workers should address the fears and rumors about sick cell disease and treatment.

*“You should address their fears and the rumors because personally, that is what made me afraid at first”* -- IDI, 4, male, adult, Mulago.

Healthcare workers should advise/encourage patients to adhere to sickle cell medication:

*“You must encourage the patient just like you would say, “You have to swallow folic acid because it increases the blood in the body, if you miss, then the blood levels reduce yet these cells need blood”. So even for that medicine, he/she must swallow it—so one must swallow it daily just as one did for folic acid”* –.

- IDI, 22, male, adult, Mulago.

There should be free drugs availed at the health facilities:

*“Another thing is medicine is expensive! So, what will encourage us to come is, we should get free medicine if it is there. But the one we used to take was easy to access; if they bring us that medicine and at least we get it, find it here, then I am certain that we won’t miss any clinic appointment. Because when you come, you expect to go back with medicine, all you must do is invest in the transport”.*

-- IDI, 14, female, caretaker, Mulago.

Healthcare managers should ensure a consistent and sufficient drug supply.

Doctors should do follow-ups of patients and set up reminder mechanisms to adhere to treatment and keep routine clinic visits.

*“Now there is a certain lady, they gave it to her and after it got finished, she stopped there claiming, “I thought you must give it to us. It is expensive in the pharmacies, and I thought I had to get it from here. Why would I buy it?” She was like, “Who is buying the medicine? Me? It is expensive! If you are giving it to us, give it to us consistently”. Other people need more teaching about the thing, and telling them, “If the child takes it, it will reduce the number of times you come here due to the child’s severe health condition” --* IDI, 28, female, Youth, Mulago.

Healthcare facilities should plan to offer transport refunds to sickle cell patients, *“Support us by giving us money for the transport fare. Sometimes we are unable to afford it”.*

*“Apart from the government supplying the medicine; maybe about the transport as well. If they can, they could organize for us some transport when we are bringing them here because these children—sometimes we get challenges, and it affects the family. We even get disagreements between the mother and father; sometimes you are supposed to take the child and he says, “I have no money! If you can go, then go. If you are unable to go, you will go some other time” yet the appointment date is due. So, those are some of the challenges we experience”.*

-- IDI, 26, female, youth, Mulago.

Health facilities should allow credit options for patients who lack funds to buy drugs.

*“What I think—okay, is it possible to give the people the drugs today and they pay for it another time; you record it down. I want them [doctors] to give us patients] the medicine on credit then they pay later”.*

--IDI, 20, male, youth, Mulago.

### Family

Financial empowerment for women to support sickle cell children at the family level should be done. Work opportunities or Income generation activity (IGA) for caregivers or sickle cell patients should be identified.

*“So, we [women] must hustle even more than men—sometimes we come and there is no medicine completely! …for our children, we should be assisted in that regard, train us in income generation activities to support our children’’*-.

-- IDI, 30, female, adult, Mulago.

### Community-based

Extension of sickle cell health services to the communities and local districts should be done to resolve the issue of transport.

*“We need health facilities in every district because sickle cell patients are quite a number. In my village I was the only one and the whole world knew that I had the ‘virus’ as they used to call it. [Hmmm] and it hindered the boy who almost married me; they told him, “Don’t bother! That one is going to die”. But now… we need those health facilities”.*

-- IDI, 27, female, adult Mulago.

*“…they could put up health facilities in communities with health workers who handle sickle cells because you leave the place in severe pain—one time my leg was in severe pain; I had not yet seen the doctor. So, if the health facility was nearby—at that time I would go to Nakaseke, and they work on me. But now, there are no health workers who handle sickle cell cases in Namuwogga”*-.

-IDI, 27, female, adult Mulago.

Sensitization of patients and community people about Hydroxyurea should be done.

*“Patients should be intensively sensitized about it [hydroxyurea] so that they can understand it very well. They should sensitize them so that they understand very well how effective it is”.*

-- IDI, 32, female, adult, Mulago.

### National

Government should reduce the price of sickle cell medicine to enhance access for all patients.

*“If it is possible—because most times the drugs are not in stock here, so they should reduce on the price or cost of the medicine…reduce the cost of that medicine. Some people cannot even start taking it; they live far away, from where they get the medicine, yet it is also expensive. So, it would hinder him or her from accessing it”.*

-- IDI, 11, Male, youth, Mulago.

The government should put up sickle cell health facilities in rural areas:

*“Government should supply the medicine to the health facilities in the rural areas. Supply the medicine for sickle cell disease because even in the government health facilities; if you want medicine, they tell you it is out of stock because I have ever gone there when my drugs were over”.*

-- IDI, 13, female, youth, Mulago.

The government could support patients by providing more drugs in the health facility or in our clinic here:

*“Government should supply the medicine to the health facilities in the rural areas. Supply the medicine for sickle cell disease because even in the government health facilities; if you want medicine, they tell you it is out of stock because I have ever gone there when my drugs were over”* -- IDI, 13, female, youth, Mulago.

There is a need for a collaborative partnership with international or non-governmental organizations to support sickle cell patients:

Now, the government; because I have ever been NTV, talking about this issue. Just like they support our friends with HIV, they should also find international organizations to support us as well. Because HIV is serious, these patients are difficult! At least they could get for us only the capsules.

-- IDI, 24, female, youth, Mulago.

## Discussion

The overall prevalence of HU use among participants who qualify for its use was high at 78%. This is the first study in Uganda to document the prevalence of HU use among patients with SCD. A literature search didn’t yield any studies documenting the prevalence of HU use among eligible patients. A Nigerian study that assessed Hydroxyurea utilization as a lesson in Public Health found that 65% of patients assessed were eligible for HU use and zero were using it, 5% of patients had been informed of or were aware of hydroxyurea as a treatment option for sickle cell disease [12]. In our study, though 88.2% of patients knew HU as treatment for SCD.

The high prevalence use of HU found in our study is unlike what other studies found in Africa, this is not uncommon given the high prevalence of SCD in these regions of the world. The high prevalence of use is explained by policies that encourage treatment of SCD e.g., HU is listed on the essential medicine list informed by the high prevalence of SCD hence it is procured and made available up to national referral hospitals. Patients are reviewed by healthcare workers in national referral hospitals who are experts in their field, and such are up to date with the latest management protocols for SCD, this is a key driver in the high prescription rate of HU.

In this study, patient-related barriers to HU use included financial constraints, poor adherence to sickle cell medication, missed routine clinic visits or appointments, psychosocial and emotional challenges, poor communication with health providers, and drug-related side effects (headaches, dizziness, frequent urination and yellowing of eyes).

To the best of our knowledge, this is the first study to document patient-related barriers to HU use among patients with SCD in Uganda. These barriers are like those reported by a cross-sectional survey done in Nigeria. Among patients and caregivers, barriers included lack of knowledge; perceived side effects; cost; religious beliefs of disease causation; and lack of pediatric formulation [13]. Another study done in Chicago, USA, looked at barriers to hydroxyurea adherence and health-related quality of life in adolescents and young adults with sickle cell disease, they found participants reported negative beliefs/motivational barriers (32%), recall barriers/forgetfulness (44%), and access barriers/paying for hydroxyurea and/or getting refills on time (32%) [14]. Another study looked at barriers to the use of hydroxyurea in the management of sickle cell disease in Nigeria, reported side effects profiles as the commonest barrier, reported concern for infertility (52.0%), and safety profile of HU in pregnancy and lactation (48.2%) [15]. A U.S. regional collaborative report on barriers to hydroxyurea use from the perspectives of providers, individuals with sickle cell disease, and families found providers and patient/caregiver reports about hydroxyurea use were inconsistent with one another; adults 26 years and older were least likely to be on hydroxyurea; and the likelihood of being on hydroxyurea decreased with one or more barriers, they also found that, even for patients on hydroxyurea, challenges to taking the medicine at the right time and forgetting were crucial unintentional barriers to adherence. Intentional barriers such as worry about side effects and “tried and it did not work” were important barriers for young adults and adults [16].

These barriers are not uncommon or unique since SCD is a chronic disease that requires daily medication. Similar challenges are experienced by other patients with chronic conditions [17]. Patients are bound to get treatment fatigued leading to poor adherence and missed appointments, get depressed, and sometimes suicidal. SCD is prevalent in the black population most of whom are residing in resource-limited settings like sub-Saharan Africa where finances are a major factor in accessing health care. Even in the US, SCD is prevalent among the black population [14] and most of these populations experience socioeconomic inequalities in developed countries.

These challenges are correlated in that financial constraints coupled with treatment fatigue led to poor adherence to treatment missed appointments, and psychosocial and emotional intrigue.

Other barriers reported were categorized as facility and these included drug stockouts, poor quality of care (referred as few health personnel to attend to them, and lack of counseling services). Community barriers included knowledge gaps and poor access to sickle cell medication in nearby health centers.

These barriers have been reported by other studies, in Nigeria, among clinicians, barriers included limited knowledge of the drug, as well as low self-efficacy to prescribe among physicians and to counsel among nurses; perceived side effects; perceived patient preference for traditional medicine; cost for patient and expense of accompanying laboratory monitoring; and limited availability of the drug and equipment for laboratory monitoring [18]. Another study in Nigeria reported barriers to hydroxyurea utilization identified by practitioners included safety and toxicity profile (100%), patient compliance (100%), effective follow-up (100%), drug availability (100%), affordability (100%) and specifically concern for reactivation of latent tuberculosis (50%) and carcinogenesis (100%) and teratogenicity (100%) [12].

In Africa, drug stockouts are a commonality, it is especially more pronounced if the medicine is used for chronic care like HU for SCD as opposed to an acute illness. Government programs support access to life-saving medications for human immunodeficiency virus (HIV), Tuberculosis (TB), and malaria; however, this is not the case for SCD. Advocacy efforts to sustain the continued availability of HU in the SCD treatment program are necessary.

Suggested recommendations by participants included; continuous advice and encouragement from health care workers, continuous sensitization about the benefits and risks of hydroxyurea to create awareness, peer to peer approach using experienced patients to support each other, the need for financial support to start up a business as a source of income for medication to meet transport, food, and other needs, self-motivation and self-love is needed among patients and urge patients to drink plenty of water and mind their diet and clothing.

These recommendations have been suggested by other studies i.e., prospective evaluation of patient’s perceptions of SCD and hydroxyurea in relation to adherence, HRQOL domains, and clinical outcomes is warranted [11]. 

Training of sickle cell care providers to attain and maintain competence in the use of hydroxyurea for the treatment of SCD was recommended by the researchers of the study on the level of utilization and provider-related barriers to hydroxyurea use in the treatment of SCD in Jos, Nigeria [19]. Researchers from an Irish study on the Irish SCD population reported that the smartphone app was expressed by the majority, with daily medication reminders being the most popular feature [11].

The suggested recommendations by participants are like interventions already in play in other public health programs such as comprehensive HIV treatment and care programs [20]. Such programs include social economic empowerment activities that equip them with skills for independent income generating for the sustenance of basic care. This allows patients to afford complementary medication, and nutrition and facilitates adherence to clinic appointments [21]. Such can be adapted into the SCD management program to offset challenges related to finances. With financial empowerment, all the other correlated challenges will be lessened.

Patients recognize the importance of medical information in advancing compliance with treatment. Programs geared towards increasing healthcare knowledge on novel SCD treatments like HU should be promoted. This will trickle down to more quality medication counseling provided to patients which will then lead to medication compliance. Expert patients could be trained to be peer influencers, by equipping them with information, educational, and communication material necessary to empower fellow patients psychosocially. This strategy has been successfully implemented in other public health challenges e.g., HIV and TB [22].

Other recommendations were health facility-based and these included; Healthcare workers should advise and encourage patients to adhere to sickle cell medication, There should be free drugs availed at the health facilities, Health managers should ensure consistent and sufficient drug supply, Health care workers should create awareness to patients about benefits of sickle cell treatment, Health care workers should offer continuous advice, health education to patients, Health care facilitates should plan to offer transport refund to sickle cell patients, Health facilities should allow credit options for patients who lack funds to buy drugs.

Community-based recommendations included the extension of sickle cell health services to the communities and local districts should be done to resolve the issue of transport, and Sensitization of patients and community people about Hydroxyurea.

Task shifting in terms of personnel and medication is necessary for grassroots accessibility. Such models have been implemented to improve life-saving therapy like ART for HIV-infected patients and disseminated drug delivery models [23]. 

National-based recommendations included the government reducing the price of sickle cell medicine to enhance access for all patients, the Government putting up sickle cell health facilities in rural areas, and there is need for a collaborative partnership with international or non-governmental organizations to support sickle cell patients.

### Study limitations

This study was carried out at national referral hospitals which have a better supply of medicines and health care experts in the management of SCD, this created selective bias because the quality of care is not generalizable to other health centers in the country. Future research directions should include national surveys to understand the magnitude of the barriers to better government planning in the allocation of resources.

Because of the Cross-sectional nature, of the quantitative section, we were not able to determine causation but could only determine the association between HU use and patient-related barriers to HU use.

### Study strengths

The study was conducted at two national referral hospitals that are in the central region of Uganda. This region has one of the highest prevalence of SCD The central region of Uganda is metropolitan with individuals coming in from different parts of Uganda so the SCD population may be representative. The findings of this study are therefore representative of the sickle cell population in Kampala which is the capital city of Uganda.

This is a mixed-method study, so the qualitative aspects of the study helped to explain the results of the quantitative findings. For example, in this study, we now know that lack of affordability is the reason why most patients have never been on HU even if it is indicated or stop using it when they need it.

## Conclusion

Implementing the use of HU has been challenging in Uganda and needs improvement. Facilitators to hydroxyurea use have been highlighted, though Patient-identified barriers at individual, facility, and community levels that need to be resolved. The experiences and insights shared by our participants provide invaluable guidance for increasing the uptake of HU. Further studies are needed to establish validated instruments to assess patients’ pain communication and adherence to the HU regimen. The prevalence of HU use among eligible patients is high at 78%.

## Data Availability

The datasets used and/or analyzed during the current study are available at https://github.com/PNamaganda/Sickle-cell-Data-in-Uganda. Deidentified data and analyzed data for this manuscript are available from the corresponding author upon request.

## Declarations

### Human protection

All data collection methods and analysis were carried out in accordance with relevant Good clinical care practice (GCP) guidelines and regulations.
